# Peripheral oxytocin is inversely correlated with cognitive, but not emotional empathy in schizophrenia

**DOI:** 10.1371/journal.pone.0231257

**Published:** 2020-04-07

**Authors:** Christiane Montag, Johanna Schöner, Lucas Guilherme Speck, Sandra Just, Frauke Stuke, Johannes Rentzsch, Jürgen Gallinat, Tomislav Majić

**Affiliations:** 1 Department of Psychiatry and Psychotherapy, Berlin Institute of Health, Charité Universitätsmedizin Berlin, corporate member of Freie Universität Berlin, Humboldt-Universität zu Berlin, Campus Charité Mitte, Berlin, Germany; 2 Department of Psychiatry, Psychotherapy and Psychosomatics, Brandenburg Medical School ‘Theodor Fontane’, Neurupppin, Germany; 3 Clinic and Policlinic for Psychiatry and Psychotherapy, University Clinic Hamburg-Eppendorf, Hamburg, Germany; Technion Israel Institute of Technology, ISRAEL

## Abstract

Endogenous oxytocin has been associated with different aspects of social cognition in healthy subjects and patients with schizophrenia. In this pilot study, we investigated the relationship between plasma oxytocin and oxytocin level changes induced by empathy-eliciting, attachment-related movie scenes with correlates of cognitive and emotional empathy in patients and healthy controls. The Multifaceted Empathy Test (MET) and the Interpersonal Reactivity Index (IRI) were administered to patients with schizophrenia (N = 35, 12 females) and healthy controls (N = 35, 12 females) to estimate dimensions of cognitive and emotional empathy. Peripheral basal oxytocin concentrations and oxytocin responses to movie-based emotional stimuli were assessed using radioimmunoassay with sample extraction. In patients, induced oxytocin level changes were inversely correlated with MET cognitive empathy regarding negative emotional states. Controlling for non-social cognition and age revealed a significant negative association between basal oxytocin levels and MET cognitive empathy for positive emotions. In healthy subjects, oxytocin reactivity was inversely correlated with the IRI subscale “fantasy”. Oxytocin was not related to any measure of emotional empathy. A hyper-reactive oxytocin system might be linked to impaired cognitive empathy as a part of a dysfunctional regulative circuit of attachment-related emotions and interpersonal stressors or threats by attribution of meaning. Healthy adults with a disposition to identify with fictional characters showed lower oxytocin reactivity, possibly indicating familiarity with movie-based stimuli. The oxytocinergic system may be involved in maladaptive coping mechanisms in the framework of impaired mentalizing and associated dysfunctional responses to interpersonal challenges in schizophrenia.

## 1. Introduction

Empathy is the ability to share, understand, and respond to the emotional state of another person, and to flexibly regulate this empathic experience [1]. Cognitive components of empathy require the capability to theoretically take the perspective of another person and to recognize or to infer their emotional mental state, while emotional empathy refers to sharing the emotional experience of another person, e. g., to vicariously feel a similar, “isomorphic” emotion [2]. In schizophrenia, deficits of cognitive empathy like emotional perspective-taking or empathic accuracy have been consistently reported [3, 4], whereas research regarding emotional empathy is less conclusive: some studies report poor emotional responsivity in patients when compared to controls [4], others suggest intact or even increased levels of emotional empathy or affective sharing in schizophrenia [1, 5]. However, as an integrative part of the social-cognitive processing system, empathy might be decisive for interpersonal functioning in this disorder [6].

A variety of human social and attachment-related behaviors are modulated by the neurohormone and neurotransmitter oxytocin (OXT) [7]. OXT may increase the salience of social cues and attenuate the autonomic and endocrine stress response, thus facilitating social approach, affiliation, and prosocial behaviors [8, 9]. In addition, OXT is critically involved in social cognition and emotion regulation [8, 10]

Despite the growing body of research surrounding schizophrenia, the delicate relationship and potential modulation of schizophrenia symptoms has not yet been fully understood; though notable interactions between dopaminergic (DA) pathways and OXT have been found in the medial prefrontal cortex, the ventral striatum, and the mesolimbic and tubero-infundibular DA pathway [11–14]. Moreover, DA receptors have been found on OXT cells in the hypothalamus [15]. Thus, and given the crucial role of DA in the pathophysiology of schizophrenia, it has been hypothesized that OXT affects symptoms of schizophrenia due to interactions between OXT and DA pathways [16, 17]. Furthermore, it has been suggested that a propensity for psychosis is intertwined via complex developmental interactions with early caregiving environments, maturation of the OXT, DA and hypothalamo-pituitary-adrenal-systems, as well as with mentalizing abilities and self-regulative function [18].

As OXT has been shown to be critically involved in prosocial and empathetic behaviour, a rich body of literature has emerged about the effects of intranasal OXT in the treatment of schizophrenia [19–22]. While some studies have highlighted a potential role of OXT in mitigating the deficits of social cognition in schizophrenia [19–22], a recent meta-analysis could not confirm a beneficial effect of OXT on negative, positive or general psychopathology [23]. Within the large body of literature concerning OXT treatment of patients with schizophrenia, there has been little investigation of the endogenous OXT system and its role in social cognition and empathy, and evidence regarding an association between endogenous OXT and social-cognitive capacity in patients is scarce and inconclusive. Few studies reported a positive relationship between baseline OXT levels and social cognitive abilities like emotion recognition from facial [24] or social cues [25] and dynamic body expressions [26], while another study showed a negative correlation between baseline OXT and theory of mind [27]. Rubin et al. [28] found correlations between peripheral OXT and perceiving faces as happier, but not with emotion recognition accuracy in women with schizophrenia. Evidence is equally scarce and contradictory with respect to OXT reactivity in response to socio-emotional stimuli: Following trust-related interactions, healthy controls displayed increased OXT levels, whereas this effect was not observed in patients with schizophrenia, with low OXT levels predicting social withdrawal [29]. In contrast, a study carried out by this work group found pronounced OXT increases in schizophrenia patients, but not in healthy controls after exposure to children’s movie scenes of attachment and loss [30]. Results of this study [30] also confirm that movie clips are a highly effective tool to elicit emotions and empathy in patients with schizophrenia as in other population [31], although few studies have examined the OXT response to emotional videos in non-clinical [32–35] or clinical populations [30] and show overall inconclusive results. In healthy women, a decrease of OXT after positive and unchanged OXT levels after negative emotions could be observed [32]. In contrast, a modest rise of plasma OXT levels in females with schizophrenia during a film’s bonding scene, but its significant decrease during an abandonment scene was reported [33]. Watching a father talk about the terminal illness of his son lead to increased peripheral OXT concentrations in healthy adults [34, 35].

In previous studies, two different measures of OXT have been taken: 1) plasma levels, reflecting trait aspects, and 2) the reactivity of the OXT system, estimated as elevation of OXT levels in response to a stimulus, relative to a basal concentration, reflecting state aspects. While the focus of our previous study [30] was the quantitative OXT reactivity after emotional movies, the objective of the present explorative study was to reach a better understanding of the underlying dynamics of the OXT system and its connection with social cognition and empathy. Therefore, we combined state and trait measures: 1) the reactivity of the endogenous OXT system to movie-based, empathy-eliciting cues, as shown in [30] and 2) baseline endogenous OXT levels. Behavioral correlates of empathy were assessed with two well-established instruments, the Multifaceted Empathy Test (MET; [36]) and the Interpersonal Reactivity Index (IRI; [37]).

The aim of this study was to assess possible associations of basal and induced OXT plasma levels with different dimensions of social cognition, like cognitive and emotional empathy, in patients with schizophrenia, extending the experiment reported by Speck et al. [30]. As no assumptions could be made about the direction of such a connection based on previous research, it was hypothesized on an exploratory basis that baseline OXT levels as well as OXT level changes in response to children’s movies of attachment and loss would be associated with cognitive and emotional empathy, and that these associations would differ between schizophrenia patients and healthy controls.

## 2. Material and methods

### 2.1. Participants

The study is an extension of the experiment reported by Speck et al. [30]. It was approved by the institutional review board “Charité’s Ethics Committee” of the Charité—Universitätsmedizin Berlin. Participants provided written informed consent. The capacity to consent was evaluated by the study team according to the principles defined by Kröber [38]. 35 in- and outpatients with paranoid schizophrenia were recruited at the PUK Charité at St. Hedwig Hospital. It should be noted that the same individuals were included as in the study by Speck et al [30]. Inclusion criteria for patients were: i) diagnosis of paranoid schizophrenia as determined by the Structured Clinical Interview (SCID) for the Diagnostic and Statistical Manual of Mental Disorders-IV (DSM-IV; Wittchen et al., 1997), administered by psychiatric research clinicians, ii) no current or lifetime Axis I psychiatric disorder except for schizophrenia spectrum disorders. The patients’ aptitude for the emotion induction experiment and symptom severity were evaluated by the treating psychiatrist. Acute suicidality, organic brain disease and current substance abuse were exclusion criteria. 35 healthy individuals participated in the study (Table 1). Healthy controls were recruited through verbal advertisement. Inclusion criteria for healthy controls were i) no current or lifetime Axis I or II psychiatric disorder as assessed by MINI-International Neuropsychiatric Interview (Sheehan et al., 1998) and SCID-II (Wittchen et al., 1997), ii) no family history of Axis I mental disorders in first- or second-degree relatives. Consumption of alcohol and cannabis 24 hours prior to testing was considered as an exclusion criterion.

**Table 1:** Demographic data, illness characteristics, basal and induced oxytocin levels and dimensions of empathy in patients with schizophrenia and healthy controls.

	Patients with schizophrenia (N = 35)	Healthy controls (N = 35)	Statistics
Gender (m/f)	23/12	23/12	^1^χ2 = 1.00
Age (mean years ± SD)	40.4 ± 8.8	36.0 ± 10.4	^2^T = 1.882
Verbal IQ	107.2 ± 18.0	114.1 ± 17.6	^2^T = -1.620
AVLT(1–5) Score	8.5 ± 2.2	10.7 ± 2.0	^2^T = -4.340***
Age at first episode (yrs.)	27.7 ± 8.9	-	-
Duration of illness (yrs.)	12.4 ± 8.4	-	-
PANSS positive score	18.2 ± 9.8	-	-
PANSS negative score	19.7 ± 7.5	-	-
PANSS general score	32.6 ± 11.0	-	-
Antipsychotic dose (CPZ, [mg])	386.4 ± 349.7	-	-
OXT baseline [pg/ml]	4.59 ± 3.35	5.49 ± 4.50	^3^U = 576.000
OXT reactivity emotion induction	1.22 ± 0.50	0.96 ± 0.38	^3^U = 371.000*
OXT reactivity control condition	1.18 ± 0.48	1.07 ± 0.55	^3^U = 509.000
MET CE (sum)	20.2 ± 4.6	22.8 ± 5.1	^2^T = -2.248*
MET CE negative valences	9.9 ± 2.7	11.0 ± 3.5	^2^T = -1.529
MET CE positive valences	10.4 ± 2.9	11.8 ± 2.3	^2^T = -2.224*
MET EE (sum)	206.9 ± 68.7	198.9 ± 51.8	^2^T = 0.495
MET EE negative valences	103.0 ± 32.8	105.2 ± 23.4	^2^T = -0.319
MET EE positive valences	103.1 ± 39.8	93.7 ± 31.4	^2^T = 1.093
IRI perspective-taking	24.1 ± 4.1	25.9 ± 3.4	^2^T = -2.017*
IRI fantasy	23.6 ± 4.7	22.6 ± 5.0	^2^T = 0.826
IRI empathic concern	25.9 ±4.7	25.7 ± 4.0	^2^T = 0.162
IRI personal distress	19.4 ± 4.6	15.8 ± 3.4	^2^T = 3.661***

N = 35/35; between-group comparisons.

^1^: χ2-Test;

^2^: T-Test for independent samples;

^3^: Mann-Whitney-U Test.

*: p<0,05;

**: p<0,01;

***: p<0.001.

Significant results are indicated in bold type. AVLT: Auditory Verbal Learning Test; CPZ: Chlorpromazine equivalent; IRI: Interpersonal Reactivity Index; MET: Multifaceted Empathy Test; CE: cognitive empathy; EE: emotional empathy; OXT: oxytocin; SD: standard deviation

Verbal intelligence was estimated with a multiple choice vocabulary test. The Auditory Verbal Learning Test (AVLT; Heubrock, 1992) assessed verbal memory, learning and executive functions. The mean scores of the first five presentations were used for analyses. Severity of current psychotic symptoms was determined by the treating psychiatrist using the Positive and Negative Syndrome Scale (PANSS; Kay et al., 1989). Overall, patients showed rather mild to moderate symptom load. Illness and medication details are reported in Table 1.

### 2.2. Baseline OXT and induction of OXT response

All experiments were carried out as described by Speck et al. [30]. To measure OXT plasma levels, a peripheral venous catheter was placed 30 min before testing. Peripheral venous blood samples were taken 4 times, before and after the presentation of movie sequences with emotional versus non-emotional contents. The first sample was used to determine OXT baseline levels. Film scenes have been shown to be highly effective in the induction of emotional states, especially when perceived as personally relevant [31]. To induce empathy and attachment-related emotions, movie scenes portraying attachment and loss, chosen from three popular children´s movies, “Bambi” (6 minutes, 37 seconds), “The Lion King” (5 minutes, 44 seconds) and “UP!” (4 minutes, 21 seconds) were presented to the subjects. All sequences displayed a short portray of an attachment relationship, ending with the death of one of the protagonists, including loss of a mother, a father and a beloved wife, respectively. In the control condition, an uncut scene from a weather documentary was presented to the subjects (3 minutes, 37 seconds). The control film was not devoid of any social content, though it gave exclusively neutral social cues. After each movie, participants were asked about how relevant the shown movies were for their own lives and how much subjective empathy and arousal they felt. All participants were asked to sit in about one-meter distance to the screen in a relaxed posture. Subjects were given a 60-minute break between conditions. Emotional and control films were balanced between individuals regarding their order of presentation.

### 2.3. Multifaceted Empathy Test (MET)

The Multifaceted Empathy Test (MET; [36]) was developed in subjects with Asperger syndrome to simultaneously measure cognitive and emotional dimensions of empathy. The modified version of the MET used in the present study was shown to be useful in differentiating cognitive and emotional empathy in schizophrenia patients [5]. A total of forty photographs depicting people in emotionally charged situations are presented, including complex positive and negative emotions. To assess 1) cognitive empathy (MET-CE), participants are required to infer the emotional state of the individual shown in the photograph (“How does this person feel?”) and to indicate the correct the mental state descriptor from a list of four alternatives by verbal responses. To assess emotional empathy (MET-EE), participants are required to rate their own, isomorphic emotional reactions (“How strongly did you feel *the same emotion* like the person in the picture?”) in response to the pictures on a 1–9 intensity scale (0 = not at all; 9 = very much).

### 2.4. Interpersonal Reactivity Index (IRI)

The 28-item Interpersonal Reactivity Index (IRI; Davis, 1983) German version: `Saarbrücker Persönlichkeitsfragebogen´ [39] is a well-validated self-report questionnaire designed to assess the following dimensions on four subscales: 1) Perspective-Taking, defined as a tendency to adopt points of view of another person and to deliberately reason about their mental states (e. g., “I believe that there are two sides to every question and try to look at them both.”), 2) Fantasy, defined as the likelihood to identify with fictional persons (e. g., “I really get involved with the feelings of the characters in a novel”), 3) Empathic Concern, defined as feelings of concern, warmth and sympathy towards others (e. g., “I am often quite touched by things that I see happen”) and 4) Personal Distress, defined as self-oriented feelings of anxiety and discomfort in response to the distress of others (e. g., “Being in a tense emotional situation scares me”). Items are rated on a 5-point Likert scale (0 = does not describe me well, to 4 = describes me very well). Ratings are summed to yield subscale scores, with higher scores indicating greater levels of empathy.

### 2.5. Blood samples

Before the first and 1 minute after the last emotional film, as well as before and 1 minute after the control condition, citrated plasma samples were taken, centrifuged immediately and frozen (-28°C).

OXT concentrations were determined by radioimmunoassay using solid phase sample extraction by Prof. Dr. Rainer Landgraf, RIAgnosis, Munich (http://www.riagnosis.com). Assay sensitivity for this method is in the 0.1 pg/ml sample range, intra- and inter-assay variability is under 10% and no significant cross-reactivity is reported. Details of extraction method, analysis and validation are reported elsewhere [40].

### 2.6. Statistical analysis

Data were analyzed using IBM SPSS Statistics 24. Normality was determined by Kolmogorov-Smirnov-Tests. Group comparisons and Spearman rank correlations were performed as indicated in the results section. OXT reactivity for each condition was defined as the quotient of ‘OXT level after film sequence’ by ‘OXT level before film sequence’.

## 3. Results

Demographic data and illness characteristics of subjects with schizophrenia are given in Table 1. Patients showed significantly lower AVLT^(1–5)^ scores than controls. No significant differences were noted regarding age, gender and verbal IQ. Information on age at first episode, duration of illness, PANSS scores and dosage of antipsychotics are provided **in** Table 1. As expected, patients with schizophrenia scored significantly lower than healthy subjects on the cognitive sum scale of the MET and on the positive affective valences’ subscale. No significant differences were observed for emotional empathy. On the IRI patients rated themselves significantly less competent in “perspective-taking”, but rather inclined to experience “personal distress” compared to controls (Table 1).

Patients and healthy controls indicated similar levels of empathy (p = 0.103), arousal (p = 0.995) and personal relevance (p = 0.370) of the emotion induction movies, but patients perceived the control condition as significantly more stressful than healthy controls (p = 0.045). For further information, see [30].

No significant group differences were revealed for baseline OXT levels and OXT reactivity when viewing the non-emotional control movie. In contrast, during the emotional experimental condition, a significant group difference appeared, with OXT level mean increases in patients, and decreases in controls [30].

**Fig 1:**
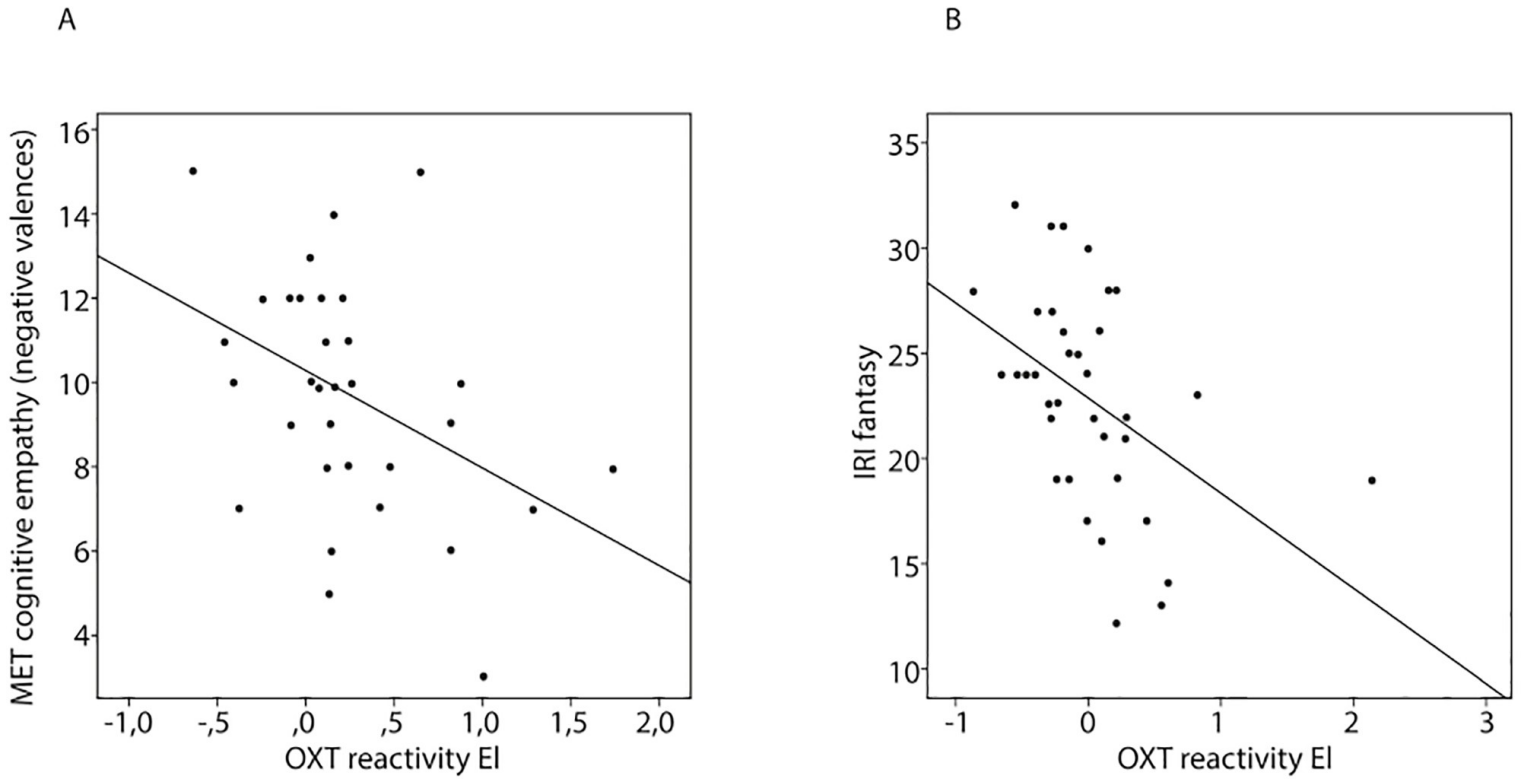
Relationship between empathy and OXT reactivity in patients with schizophrenia and healthy controls. A: Correlation between MET cognitive empathy (negative valences) and OXT reactivity in patients with schizophrenia. MET: Multifaceted Empathy Test; OXT: oxytocin; EI: emotion induction. B: Correlation between IRI “fantasy” and OXT reactivity in healthy adults. IRI: Interpersonal Reactivity Index; OXT: oxytocin; EI: emotion induction.

In patients, MET Cognitive empathy for negative emotional valences was inversely correlated with OXT reactivity in the emotional condition (Fig 1A; r = -0.418; p<0.05). This correlation coefficient differed significantly from the respective measure in the control group (Fisher’s Z = -1.982, p>0.05). No other associations were found between basal OXT, OXT reactivity and other measures of empathy in patients (Tables 2 and 3).

**Table 2:** Spearman correlation coefficients for associations between basal / induced oxytocin levels and MET cognitive and emotional empathy in patients with schizophrenia and healthy controls.

	MET CE (sum)	MET CE negative valences	MET CE positive valences	MET EE (sum)	MET EE negative valences	MET EE positive valences
**Patients**						
OXT baseline	-0.301	-0.146	-0.316	-0.056	-0.131	0.030
OXT reactivity EI	-0.280	-0.418*	-0.121	0.117	0.008	0.093
OXT reactivity Con	0.140	-0.110	0.276	0.206	0.121	0.251
**Healthy Controls**						
OXT baseline	0.023	0.077	-0.003	-0.139	-0.129	-0.107
OXT reactivity EI	0.024	0.050	-0.030	0.046	-0.036	0.075
OXT reactivity Con	-0.202	-0.279	0.008	-0.003	0.167	-0.128

N = 35/35;

*: p<0,05;

**: p<0,01;

***: p<0.001.

Significant results are indicated in bold type. Con: control condition; CE: cognitive empathy; EE: emotional empathy; EI: emotion induction; MET: Multifaceted Empathy Test; OXT: oxytocin.

**Table 3:** Spearman correlation coefficients for associations between basal / induced oxytocin levels and dimensions of empathy assessed by the Interpersonal Reactivity Index (IRI) in patients with schizophrenia and healthy controls.

	IRI ‘fantasy’	IRI ‘perspective taking’	IRI ‘empathic concern’	IRI ‘personal distress’
**Patients**				
OXT baseline	-0.216	-0.102	-0.320	0.108
OXT reactivity EI	-0.048	0.122	-0.083	-0.122
OXT reactivity con	0.147	0.176	0.099	-0.166
**Healthy controls**				
OXT baseline	0.041	-0.033	-0.173	0.064
OXT reactivity EI	-0.537**	-0.202	0.147	0.057
OXT reactivity con	-0.359*	-0.170	0.235	-0.272

N = 35/35;

*: p<0,05;

**: p<0,01;

***: p<0.001.

Significant results are indicated in bold type. Con: control condition; EI: emotion induction; IRI: Interpersonal Reactivity Index; OXT: oxytocin.

In healthy subjects, neither baseline OXT levels nor OXT reactivity were correlated with any of the behavioral measures of cognitive empathy as assessed with the MET (Table 2). However, significant inverse correlations between OXT reactivity in the emotional (r = -0.537; p<0.01) as well as in the control (r = -0.359; p<0.05) condition and the IRI dimension “fantasy” were found in healthy subjects, but not in patients (Fig 1B; Table 3). Both correlation coefficients differed significantly from the respective coefficients in the patients’ group.(emotional condition: Fisher’s z = -2.208, p>0.05; control condition: Fisher’s z = -2,095, p>0.05).

In neither group, significant associations between OXT measures and age, verbal IQ or AVLT scores were found (p>0.05). However, as social cognition is partially dependent on general cognition [41], and as in our samples MET-CE and IRI “fantasy” and “perspective-taking” were significantly related to verbal IQ, AVLT and age (see supplementary data S1 Table), additional partial correlation analyses were run with verbal IQ, AVLT and age as control variables. The significant inverse associations between MET-CE for negative valences and OXT reactivity in the emotional condition in the patient group (r = -0.407, p<0.05) and between IRI “fantasy” and OXT reactivity in the emotional condition in healthy controls (r = -0.435, p<0.05) were confirmed. The association between “fantasy” and OXT reactivity in the control condition in healthy persons was not maintained.

Moreover, introducing verbal IQ, AVLT and age as control variables to the analysis revealed another association in schizophrenia patients: Basal OXT levels were significantly and inversely associated with MET-CE for positive emotional valences (r = -0.468, p<0.01).

Baseline OXT was correlated to OXT reactivity in the emotional condition in patients (r = -0.371, p<0.05). Introducing baseline OXT as a control variable did not alter results in patients (OXT reactivity emotional condition x MET CE negative emotions: r = -455, p<0.01; OXT reactivity emotional condition x MET CE sum score: r = -0.422, p<0.05) or in healthy controls (OXT reactivity emotional condition x IRI fantasy: r = -0.498, p<0.01). Spearman’s rank correlation analysis did not show significant relations between OXT measures and self-rated empathy, arousal and personal relevance of the movie clips, antipsychotic dose, duration of illness, age at first manifestation or PANSS positive and negative scores (n = 32). However, Spearman’s rank correlation analysis did show a negative correlation between MET CE sum score and PANSS “general psychopathology” (r = -0.39, p = 0.026) and a positive correlation between MET CE sum score and medication with atypical antipsychotics (r = 0.351, p = 0.039). After including medication and PANSS “general psychopathology” as control variables, the correlation between OXT reactivity and MET-CE for negative valences remained stable (r = -0.407, p = 0.043).

Of note, using an exploratory approach, no mathematical correction for multiple testing was made.

## 4. Discussion

In this exploratory study investigating the relationship between behavioral correlates of empathy and oxytocin (OXT) in schizophrenia, endogenous OXT level changes induced by emotional children’s movies were inversely correlated with MET cognitive empathy for negative emotional valences in patients. Cognitive empathy for positive valences and all measures of emotional empathy appeared unrelated to OXT reactivity in patients. Results were more pronounced when basal OXT was used as a control variable. When variance attributable to non-social cognitive function and age was controlled for, findings remained stable. An additional significant inverse association between baseline OXT and cognitive, but not emotional, empathy for positive valences appeared. These correlations were found in patients only, whereas in healthy subjects, neither MET cognitive nor emotional empathy were associated with peripheral OXT. However, in healthy individuals, OXT level changes induced by the emotional stimuli were inversely related to the IRI subscale “fantasy”.

Behavioral results are in line with previous reports of impaired cognitive, but maintained emotional empathy in schizophrenia patients and higher degrees of personal distress in empathy-eliciting situations compared to healthy controls [3, 5]. In addition to the group differences in OXT reactivity discussed by Speck et al. [30], the present findings suggest that schizophrenia patients with an impaired ability to infer emotional mental states might be characterized by higher amplitudes of their OXT response towards empathy-inducing stimuli and by higher baseline OXT levels. Cognitive empathy, i. e. the ability to mentalize, is specifically essential for successful self-regulation, and can switch to automatic, action-based processing of social information under conditions of high arousal [42]. In schizophrenia, impairments of cognitive mentalizing might therefore be part of a vicious circle, consisting of misinterpretation of social situations, heightened interpersonal distress and emotional dysregulation, and a further compromise of social cognitive function that may result in delusional symptoms or social withdrawal. Dysfunctional emotion regulation has been linked to hyper-reactivity of the OXT system; evidence from other healthy as well as clinical populations suggests that relative increases of induced OXT levels indicate emotional and attachment-related vulnerability [43–45]. In schizophrenia, a higher prevalence of insecure attachment representations [46], higher levels of personal distress in social situations [3], contagion with negative emotions [5], dysfunctional emotion regulation strategies [47] and an impaired mentalizing capacity [41] have been consistently reported. The association of higher OXT reactivity with impaired cognitive mentalizing in our study might thus represent a physiological correlate of a dysfunctional regulation of intense, attachment-related emotions by attribution of meaning.

A similar line of thought stems from stress research. In schizophrenia, impaired tolerance to distress has been consistently associated with different stages of the illness [48] and with the exacerbation of psychotic symptoms [49]. Maternal stress and childhood adversity [50] are risk factors for the illness as such [51]. Meta-analytic findings suggest that intranasal oxytocin significantly reduces the cortisol response to stressful stimuli among different psychiatric populations [52]. There is converging evidence that OXT plays a critical role as a moderator of the stress response [8, 53, 54], possibly buffering the impact of aversive mental content during emotional processing [54] and boosting recovery from stress [55]. Although measures of the autonomous stress response were not observed in our study, it can be discussed whether the induction of negative emotions by dramatic emotional movie scenes or even the anticipation of stress or novelty [53] lead to a pronounced physiological stress response and concomitant OXT release in schizophrenia patients with more severe impairments of cognitive mentalizing.

Moreover, the present finding is partially in keeping with Walss-Bass et al. [27], who reported highest basal OXT levels in patients with delusions compared to non-delusional or healthy persons and a negative correlation between plasma OXT and social-cognitive capacity in the delusional subgroup. The authors suggested OXT to promote self-referential bias in patients with delusions or, alternatively, an increased OXT secretion in response to distress related to social-cognitive bias. In the study by Brown et al. [56], higher baseline OXT was associated with schizophrenia patients’ greater avoidance of angry faces in an Approach-Avoidance Task, indicating OXT effects specific for the interpretation of threatening emotions up to paranoid thinking. Crespi [57] supposed an integrative social-evolutionary framework including a role of OXT in both positive, fitness-enhancing, as well as negative social situations. Of note, in threatening social circumstances OXT secretion may be related to an increase in distress and anxiety to motivate coping attempts. If positive and negative social challenges can be resolved, plasma OXT may decline again due to negative feedback regulation,—if not, a hyper-reactive OXT system might continue to support social vigilance and mentalizing. Psychotic-affective conditions might therefore be characterized by hypermentalizing, high OXT levels and loss of regulatory feedback control of oxytocinergic modulation for social cognition, while autistic traits are linked to lower endogenous OXT and hypomentalizing [57]. However, in contrast to previous studies [27, 56], positive and negative symptom expression did not correlate with OXT levels in this study. MET scores do not differentiate between hyper- or hypomentalizing, and future studies should include tests that allow for a distinction of mentalizing error types [58].

Higher OXT levels at baseline were found to be associated with an impaired attribution of positive, but not negative, emotions in our study. Evidence regarding valence-specific effects of OXT is mixed in healthy [59, 60] and psychotic individuals [19, 24, 28, 61], but this might be reconcilable with the idea of OXT mediating any situation of social salience [9, 57]. As effects of OXT may be dependent on whether the environment is interpreted as positive or safe, vs. negative or threatening [57, 62], it could be speculated that in the present sample different mechanisms lead to differential, valence-specific associations of OXT with cognitive empathy: High “tonic” baseline OXT levels together with impairments to attribute positive emotions might be linked to chronic emotional vulnerability or even depression that may lead to a failure to experience reward in positive social situations. In this respect, undetected depressive comorbidity might have played a critical role, as poor recognition of MET positive emotions has been associated with depressive symptomatology [5]. It seems possible that a hyperactive OXT system in response to negative social stimuli might correspond to dysfunctional coping in threatening or stressful social environments, particularly in individuals who are unable to mentalize negative emotions.

In our study, no relationship between the endogenous OXT and the broader dimensions of empathy on the IRI subscales was found in patients. Surprisingly, a self-reported tendency to identify with fictional characters was inversely correlated with OXT reactivity in healthy controls, but not in patients. Interestingly, in a previous study, IRI “fantasy” scores were associated with subclinical delusional ideation in unaffected first-degree relatives of patients with schizophrenia [63]. Considering the assumptions of Crespi [57], the direction of the correlation with lower OXT in “high-fantasizers”- speaks against the presence of a subclinical psychosis risk in these individuals. In contrast, individuals reporting a strong tendency to resonate with characters from movies and fiction showed better non-social cognitive function. From these reports one can presume a higher familiarity with the presented emotional movies or cinema in general, and OXT responses might be lower after habituation [53, 54]. Unfortunately, study participants were not asked whether the movies were familiar to them. Alternatively, healthy controls reporting low fantasy might have answered the IRI in a sense of a habitual repression of emotional experience, but still exhibited–an even more pronounced- vegetative response to emotional contents. Our result differs from recent findings of more prominent, salivary OXT increases in empathy-biased, but not in systemizing-biased, healthy individuals during video-based induction of empathy that were weakly correlated with IRI ‘perspective taking’ [36]. This discrepancy might be attributable to lower statistical power in our study and the fact that systemizing was not assessed in our study. Moreover, children’s movies might elicit a different spectrum of emotions compared to watching a father talking about his severely ill child.

This study suffers a number of further limitations. Due to the cross-sectional and correlational experimental design, no conclusions can be drawn regarding causalities. Future studies should include detailed measures of hyper- or hypomentalizing ratings for confounding symptoms like depression and physiological measures of the stress response together with OXT levels should be taken. Temporal dynamics of OXT secretion in response to positive and negative socio-emotional stimuli should be accounted for by more frequent measurements. Of course, the question of whether peripheral OXT levels reflect central processes is still debated, though some studies suggest some coordination, particularly under experimentally induced stress [64].

Overall, although it is tempting to speculate that OXT might be part of a dysfunctional regulative circuit of attachment-related emotions and interpersonal stressors, it should be acknowledged that this is only one possible explanation for our findings; the whole picture needs to be completed by further studies.

Although sex-specific interactions between OXT and social cognition have been previously described [28], gender and sexual hormones were not a focus of this study and therefore not included as possible mediators in our analysis. Another source of uncertainty is the heterogeneity of our patient sample. However, in order to take account of the possible influence of symptom load and illness characteristics, we included overall psychopathology and medication as confounders.

Furthermore, it is important to acknowledge that there is no established standard protocol in the measurement of OXT after viewing movie clips in German language. The experimental setup was based on the few other studies that have investigated OXT response to video-based or real-life stimuli [29, 33, 35]. Nonetheless, a systematic understanding of the physiological OXT response to emotional stimuli in healthy adults is still lacking, and it could be argued that introducing a new paradigm to compare patients with controls might lead to a reduced interpretability. Although the importance of the endogenous OXT as a predictor of social cognitive function has recently been stressed [65], future research is necessary to shed light on endogenous OXT baseline levels and reactivity in healthy individuals as well as in clinical populations in order to achieve a higher comparability. Moreover, it is unclear, whether passively viewing a movie elicits the same level of emotions and hormonal reactivity as an active social interaction. The measurement of emotional and cognitive empathy was not based on the reactions to the movie paradigm, and self-rated empathy, arousal and personal relevance after the film clips did not correlate with OXT responses in either group [30]. This might be due to a discrepancy between autonomous arousal and experiential aspects of emotion in patients [66], or the fact that personally salient aspects of the films, evoking various emotions or memories, might have differed individually. Standardized empathy tests and questionnaires might capture dimensions of empathy more specifically, while OXT responses rather reflect a general emotion regulation process. Of note is that a newer study by Procyshyn et al [34] reported an association between video-induced OXT level changes and a healthy individuals’ individual dispositions towards empathizing, thus indicating a possible link between neurobiological changes during passive viewing and dispositional aspects of empathy.

Main limitations are caused by small case numbers and reduced statistical power. This pilot study was lead under exploratory premises, and alpha-levels were therefore not adjusted for multiple testing. We are aware that the findings of the present study should thus be interpreted with caution. Further studies with larger sample sizes are certainly required to highlight the complex interplay between social cognition and the endogenous OXT-system in patients with schizophrenia.

## 5. Conclusion

In summary, our findings corroborate a potential role of OXT in deficits of cognitive empathy in schizophrenia. A hyper-reactive OXT system might be an index for an unsuccessful coping with positive and negative interpersonal challenges [57]. Research regarding the therapeutic application of OXT might therefore consider the individual reactivity of the OXT system, and also focus the impact of non-pharmacological interventions to improve mentalizing, stress tolerance and emotion regulation on the endogenous OXT system.

## Supporting information

S1 Table Correlation between cognitive empathy and confounders. (DOCX)
