# Supplementary material for: Peripheral oxytocin is inversely correlated with cognitive, but not emotional empathy in schizophrenia
Source: PLoS One. 2020 Apr 7;15(4):e0231257. doi: 10.1371/journal.pone.0231257 (PMC7138301; doi:10.1371/journal.pone.0231257)
Supplement: S1 Table — (DOCX) [file pone.0231257.s001.docx]

Supplementary data S1: **Correlation between cognitive empathy and confounders.**

1. **Total Sample**

|  | Cognitive Empathy (MET) | IRI fantasy | IRI perspective taking |
| --- | --- | --- | --- |
| Age | -0.124 | -0.235* | -0.259^*^ |
| IQ (MWT-B) | 0.548^**^ | 0.109 | 0.063 |
| AVLT mean score | 0.398^**^ | 0.362^**^ | 0.452^**^ |
| MET: Multifaceted Empathy Test, IQ: Intelligence Quotient; MWT-B: Mehrfach-Wortschatz-Intelligenz-Test B; AVLT: Auditiver verbaler Lerntest; IRI: Interpersonal Reactivity Index | | | |
| **. p<0.01. | | | |
| *. p<0.05. | | | |

1. **Schizophrenia patients**

|  | Cognitive Empathy (MET) | IRI fantasy | IRI perspective taking |
| --- | --- | --- | --- |
| Age | 0.078 | -0.190 | -0.101 |
| IQ (MWT-B) | 0.494** | 0.166 | -0.080 |
| AVLT mean score | 0.377^*^ | 0.451** | 0.297 |
| MET: Multifaceted Empathy Test, IQ: Intelligence Quotient; MWT-B: Mehrfach-Wortschatz-Intelligenz-Test B; AVLT: Auditiver verbaler Lerntest; IRI: Interpersonal Reactivity Index | | | |
| **. p<0.01. | | | |
| *. p<0.05. | | | |

1. **Healthy Controls**

|  | Cognitive Empathy (MET) | IRI fantasy | IRI perspective taking |
| --- | --- | --- | --- |
| Age | -0.180 | -0.325 | -0.346* |
| IQ (MWT-B) | 0.555^**^ | 0.100 | 0.141 |
| AVLT mean score | 0.274 | 0.479^**^ | 0.531** |
| MET: Multifaceted Empathy Test, IQ: Intelligence Quotient; MWT-B: Mehrfach-Wortschatz-Intelligenz-Test B; AVLT: Auditiver verbaler Lerntest; IRI: Interpersonal Reactivity Index | | | |
| **. p<0.01. | | | |
| *. p<0.05. | | | |
